# Environmental Chlamydiae Alter the Growth Speed and Motility of Host Acanthamoebae

**DOI:** 10.1264/jsme2.ME11353

**Published:** 2012-10-26

**Authors:** Miho Okude, Junji Matsuo, Shinji Nakamura, Kouhei Kawaguchi, Yasuhiro Hayashi, Haruna Sakai, Mitsutaka Yoshida, Kaori Takahashi, Hiroyuki Yamaguchi

**Affiliations:** 1 Department of Medical Laboratory Science, Faculty of Health Sciences, Hokkaido University, Kita-12, Nishi-5, Kita-ku, Sapporo, Hokkaido 060–0812, Japan; 2 Division of Biomedical Imaging Research, Juntendo University Graduate School of Medicine, 2–1–1 Hongo, Bunkyo-ku, Tokyo 113–8421, Japan; 3 Division of Ultrastructural Research, Juntendo University Graduate School of Medicine, 2–1–1 Hongo, Bunkyo-ku, Tokyo 113–8421, Japan

**Keywords:** symbiosis, environmental chlamydiae, *Protochlamydia*, *Neochlamydia*, *Acanthamoeba*

## Abstract

Symbiosis between living beings is an important driver of evolutionary novelty and ecological diversity; however, understanding the mechanisms underlying obligate mutualism remains a significant challenge. Regarding this, we have previously isolated two different *Acanthamoeba* strains harboring endosymbiotic bacteria, *Protochlamydia* (R18 symbiotic amoebae: R18WT) or *Neochlamydia* (S13 symbiotic amoebae; S13WT). In this study, we treated the symbiotic amoebae R18WT and S13WT with doxycycline (DOX) and rifampicin (RFP), respectively, to establish the aposymbiotic amoebae R18DOX and S13RFP, respectively. Subsequently, we compared the growth speed, motility, phagocytosis, pinocytosis, and morphology of the symbiotic and aposymbiotic amoebae. The growth speed of R18DOX was decreased, although that of S13RFP was increased. A marked change in motility was observed only for R18DOX amoebae. There was no difference in phagocytic and pinocytic activities between the symbiotic and aposymbiotic amoebae. Meanwhile, we observed a significant change in the phalloidin staining pattern and morphological changes in R18DOX (but not S13RFP) aposymbiotic amoebae, indicating a change in actin accumulation upon removal of the *Protochlamydia*. Infection of C3 (a reference strain) or S13RFP amoebae with *Protochlamydia* had a harmful effect on the host amoebae, but R18DOX amoebae re-infected with *Protochlamydia* showed recovery in both growth speed and motility. Taken together, we conclude that endosymbiont environmental chlamydiae alter the growth speed and/or motility of their host *Acanthamoeba*, possibly implying an close mutual relationship between amoebae and environmental chlamydiae.

Bacterial endosymbiosis is a widespread and sometimes obligate relationship in insects and protozoa (32). Understanding this phenomenon may provide new insights into the evolution and ecology of host-parasite relationships (32). Symbiosis has been shown to be beneficial to hosts such as aphids, tsetse flies, ants, and worms, where the bacterial endosymbionts provide essential amino acids (7), vitamin B complexes (31), and fatty acids (35). Symbiosis has also been shown to play an essential role in oogenesis and development (6). Genomic comparisons of bacterial endosymbionts and their hosts have shown high levels of recombination in bacterial endosymbionts (34) to adapt to the host and possibly modify their metabolic pathways to complement specific aspects of host physiology and ecology (28); however, exploring the molecular interactions of symbiosis and understanding the mechanisms underlying obligate mutualism remain a significant challenge.

*Acanthamoeba* amoebae are readily isolated from a wide range of natural environments, such as soil, river water, domestic tap water, seawater, pond water, and dust (8, 20, 21). Endosymbiotic bacteria have been detected in *Acanthamoeba*, with a possibly stable interaction, although amoebae possess a powerful bactericidal mechanism (4, 9, 11, 15–17, 23, 25, 30). Investigations of *Acanthamoeba* are facilitated by the fact that these organisms grow easily and rapidly in simple media regardless of the presence of bacterial endosymbionts. They are therefore a good model system to investigate stable interactions between endosymbionts and amoebae, and to advance our understanding of the evolution of symbiosis.

Previously, we isolated environmental *Acanthamoeba* infected with endosymbiotic bacteria, and successfully established models of environmental *Acanthamoeba* persistently infected with *Protochlamydia* (R18 symbiotic amoebae: R18WT [wild type]) or *Neochlamydia* (S13 symbiotic amoebae: S13WT) (23, 25). These endosymbionts have been assigned to the order *Chlamydiales* (14), which includes *Chlamydia trachomatis* and *Chlamydophila pneumoniae*, so-called pathogenic chlamydiae that are linked to sexually transmitted disease (19) and respiratory infection (16, 19), respectively. The endosymbionts have been designated environmental chlamydiae that are also potential human pathogens associated with pneumonia or abortion (4).

In this study, to investigate the effects on amoebae of endosymbionts, we established aposymbiotic amoebae from wild-type amoebae harboring endosymbiotic bacteria (R18WT and S13WT amoebae) by treatment with antibiotics. We compared the phenotypic properties between symbiotic and aposymbiotic amoebae, including growth speed, motility, phagocytosis, pinocytosis, phalloidin staining pattern, and morphology.

## Materials and Methods

### Amoebae

As described previously (23, 25), two different amoeba strains, R18WT and S13WT, which were persistently infected with *Protochlamydia* endosymbiont (eR18) or *Neochlamydia* (eS13), were isolated from a river water sample and a soil sample, respectively. We have previously confirmed that the prevalence of amoebae with endosymbionts, as defined by 4′,6-diamidino-2-phenylindole (DAPI) staining, was always approximately 100% (for both R18WT [26] and S13WT [data not shown]). *A. castellanii* C3 (ATCC 50739) (C3 amoebae), purchased from the American Type Culture Collection (Manassas, VA, USA), was used as a reference strain.

### Bacteria

R18WT amoebae harboring eR18 *Protochlamydia* were harvested and disrupted by freeze-thawing. After centrifugation at 180×*g* for 5 min to remove cell debris, the bacteria were concentrated by high-speed centrifugation at 800×*g* for 30 min. The bacterial pellet was resuspended in sucrose-phosphate-glutamic acid buffer containing 0.2 M sucrose, 3.8 mM KH_2_PO_4_, 6.7 mM Na_2_HPO_4_, and 5 mM L-glutamic acid (pH 7.4), and then stored at −80°C until needed. The number of infectious *Protochlamydia* progeny was determined with an amoeba-infectious unit assay, using a co-culture of the amoebae established previously (22).

### Establishment of aposymbiotic amoebae

Two different symbiotic amoebae, R18WT and S13WT, were treated with the antibiotics doxycycline (DOX; 64 μg mL^−1^) and rifampicin (RFP; 64 μg mL^−1^), respectively, for 9 days, with a change of medium containing fresh antibiotics every third day. Successful elimination of endosymbiotic bacteria from amoebae was confirmed using reverse transcription-PCR (RT-PCR), fluorescent *in situ* hybridization (FISH), and DAPI staining. Aposymbiotic amoebae derived from R18WT and S13WT were designated R18DOX and S13RFP, respectively. We used different antibiotics to eliminate the two bacterial endosymbionts because of their different antibiotic susceptibilities.

### Assessment of amoebal growth

According to methods described previously (22), the amoebae were seeded into 24-well culture plates at a density of 1–5×10^4^ cells well^−1^ and cultured in PYG (peptone, yeast extract, and glucose) medium at different temperatures (15, 30, or 37°C) for up to 7 days. The number of amoebae was also determined by the trypan blue exclusion method and phase-contrast microscopy, as described previously (25).

### FISH

FISH was performed as previously reported (23). In brief, amoebae were fixed and hybridized with Alexa Fluor 488-labeled EUK 516 (green), which targets eukaryotic 18S rRNA (5′-ACCAGACTTGCCCTCC-3′) (1), or Alexa Fluor 532-labeled C_22_-658 (red), which is specific to the *Parachlamydiaceae* family (5′-TCCATTTTCGTCTAC-3′) (10). Hybridizations were performed for 90 min at 46°C. The amoebae subjected to FISH were observed with a confocal laser microscope.

### RT-PCR

Amoebae (1×10^5^) were harvested by centrifugation at 1,600×*g* for 30 min. The pellets were then used for RNA extraction, which was performed with an RNeasy Mini Kit (Qiagen, Valencia, CA), according to the manufacturer’s instructions. The extracted total RNA was treated with DNase (Ambion, Austin, TX). The amount and purity of the extracted RNA was determined using a spectrophotometer. The resulting RNA preparations were assayed for DNA contamination by PCR omitting the RT step. RT of 2 μg total RNA using avian myeloblastosis virus reverse transcriptase was performed with random primers in a commercial reaction mixture (Reverse Transcription System; Promega, Madison, WI). Template cDNA (2 μL) was used for PCR. *Acanthamoeba* and the order *Chlamydiales* were detected by conventional PCR that targeted the *Acanthamoeba* 18S rRNA (29) and *Chlamydiales* 16S rRNA (5), respectively. The amplified products were separated by agarose gel electrophoresis and visualized by ethidium bromide staining.

### Assessment of amoebal motility

We compared the motility of symbiotic amoebae (R18WT and S13WT) with that of aposymbiotic amoebae (R18DOX and S13RFP). Briefly, amoebae were seeded into 24-well culture plates at a density of 5×10^5^ cells well^−1^ and cultured in PYG for 3 h at 30°C. After incubation, the amoeba layers were scratched with a pipette tip, washed carefully with Page’s amoeba saline (PAS) (27), and allowed to migrate in PYG for up to 3 h. At 0, 1, 2, and 3 h post-incubation, the aspect of the amoebae in each observed area was captured as a digital image. Finally, the motility of the amoebae was expressed as the number of amoebae in the observed area.

### Pinocytic and phagocytic activity

Pinocytosis was measured as described previously (2). In brief, amoebae (1×10^6^ cells well^−1^) were seeded and, after 2 h incubation at 30°C, cultured in PYG with fluorescein isothiocyanate (FITC)-dextran (MW 8,000; Sigma, St Louis, MO) at a final concentration of 0.2 mg mL^−1^ for up to 200 min. The reaction was stopped by twice washing with ice-cold PAS and the amoebae were lysed by the addition of 0.2% Triton-X PAS solution. Fluorescence was measured on a luminescence spectrometer (Perkin-Elmer Corp, Waltham, MA) with an excitation wavelength of 485 nm and an emission wavelength of 535 nm. The phagocytic activity of amoebae with FITC-labeled beads (diameter 2.0 μm; excitation 470 nm, emission 505 nm; Sigma) was also assessed. Amoebae (5×10^7^ cells well^−1^) were cultured in PAS with FITC beads (approximately 5×10^8^) at 30°C for up to 50 h. The amoebae were collected for assessment of phagocytic activity after 0, 8, 24, and 48 h of incubation. The proportion of amoebae containing beads was estimated under a fluorescence microscope by observing three to five randomly selected fields-of-view containing more than 100 cells.

### Phalloidin staining and image data processing

Changes in the phalloidin staining patterns showing the localization of actin between symbiotic and aposymbiotic amoebae were determined using a fluorescence microscope and phalloidin staining according to the manufacturer’s protocol (Sigma). In brief, 1×10^6^ amoebal cells attached to a cover slip were fixed with 4% paraformaldehyde in PAS and then incubated with FITC-phalloidin (final concentration 1 μg ml^−1^) for 40 min at room temperature. Cell images were then captured and processed using the following method with Image J software (Scion, Gaithersburg, MO). The captured images were converted to gray-gradual signals with the same threshold, the cells were outlined, and the averaged gray density within each outlined amoeba was compared between symbiotic and aposymbiotic amoebae. The density within the outline was estimated by manually scanning three to five randomly selected fields-of-view containing more than 20 cells per field in at least three independent experiments.

### Morphological analysis with a scanning electron microscope (SEM)

According to a previous method (3), amoebae were washed in saline, fixed with 2.5% (v/v) glutaraldehyde in phosphate-buffered saline (pH 7.4) for 2 h at room temperature, and subsequently soaked in 2% (w/v) osmium tetroxide for 1 h at 4°C. The samples were then dehydrated in ethanol, freeze-dried, and coated with osmium using a plasma osmium coater. Ultimately, the samples were analyzed using a Hitachi S-4800 scanning electron microscope (Hitachi, Tokyo, Japan).

### Infection of amoebae (C3, S13RFP, R18DOX) and restructuring of symbiotic amoebae

A 24-well plate with C3, S13RFP, or R18DOX amoebae (cells well^−1^) suspended in PYG broth was incubated with 5×10^6^ *Protochlamydia* (multiplicity of infection of 10) for 1 h. After washing, the cultures were re-suspended in fresh medium and incubated for 7 days at 30°C in a normal atmosphere. During the culture, cells were regularly collected to determine the cell number, motility, pinocytosis, and phagocytic activity, as described above.

### Statistical analysis

Data obtained from the *in vitro* experiments were compared using Student’s *t*-test with (Fig. 7B and C only) or without a Bonferroni correction. *P*<0.05 was considered significant.

## Results

### Successful elimination of endosymbiotic bacteria from amoebae by treatment with antibiotics

**Fig. 1:**
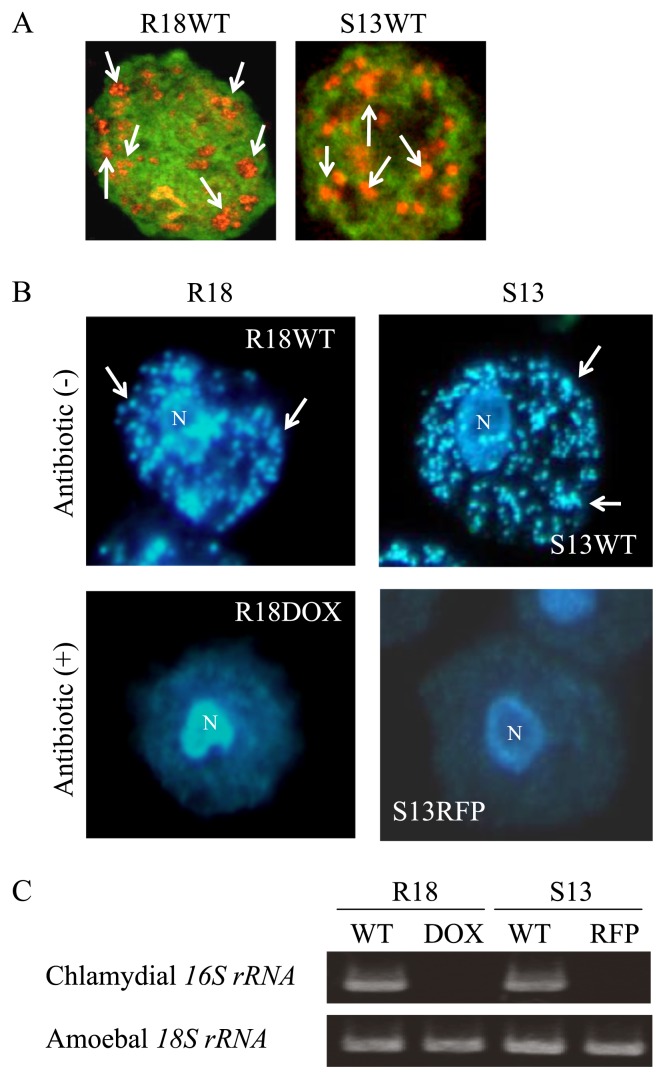
Establishment of aposymbiotic amoebae, R18DOX and S13RFP, from R18WT and S13WT, respectively, by antibiotic treatment. (A) Representative FISH images of R18WT and S13WT amoebae before treatment with antibiotics. The amoebae were observed with a confocal laser microscope. Green, amoebal 18S rRNA; orange, endosymbiotic bacteria. 1,000× magnification. Arrows show representative bacterial clusters. (B) Representative DAPI-stained images of R18 and S13 amoebae before and after treatment with antibiotics. 1,000× magnification. The amoebae were observed with a conventional fluorescence microscope. Arrows show representative bacterial clusters. N, amoebal nucleus. (C) Representative RT-PCR products amplified from amoebae and resolved by agarose gel electrophoresis. WT, wild type; DOX, doxycycline; RFP, rifampicin.

Figure 1A is a representative FISH image showing the amoebae before treatment with antibiotics, indicating that the bacterial endosymbionts localized to the cytoplasm of the amoebae, as described previously (23, 25). After antibiotic treatment, successful elimination of the endosymbionts was confirmed by DAPI staining (Fig. 1B) and RT-PCR (Fig. 1C). The established aposymbiotic amoebae were stably maintained for at least five months, exhibiting no contamination by free-living bacteria in culture. The C3 amoebae were also treated with each antibiotic, which caused no change in their phenotypic properties (growth rate, motility, phagocytosis, pinocytosis, or morphology) (data not shown). Thus, the working concentration of the antibiotics did not have any cytotoxic effect on the amoebae.

### Growth speed of symbiotic and aposymbiotic amoebae

**Fig. 2:**
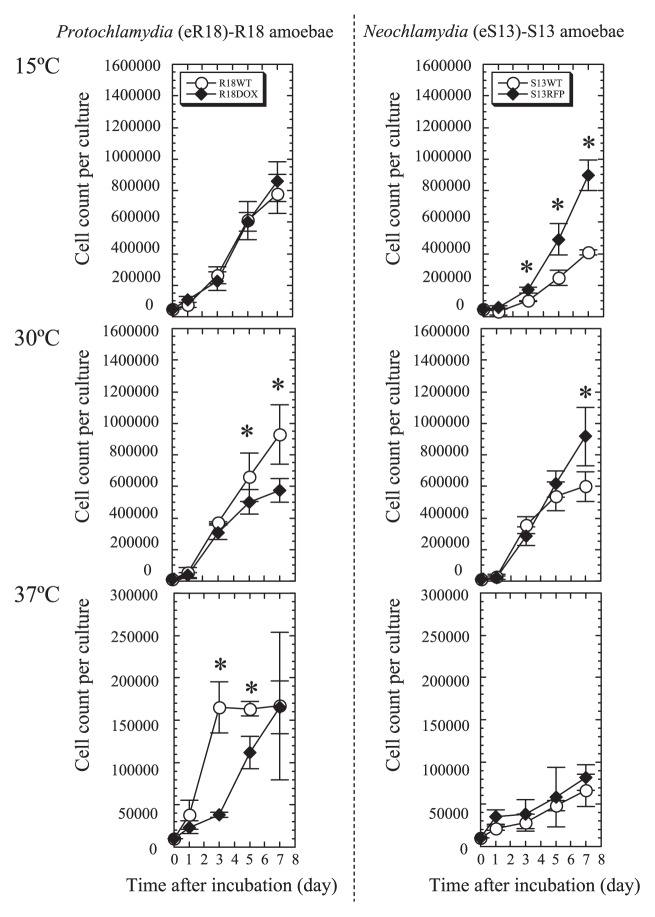
Growth speed of symbiotic amoebae (R18WT and S13WT) and established aposymbiotic amoebae (R18DOX and S13RFP). The amoebae were seeded into 24-well culture plates at a density of 1–5×10^4^ cells well^−1^ and cultured in PYG medium at different temperatures (15, 30, or 37°C) for up to 7 days. The data show the mean ± SD of at least three independent experiments. Left panels, R18 amoebae. Right panels, S13 amoebae. *P<0.05 *vs*. cultures with symbiotic amoebae (Student’s *t*-test).

*Acanthamoeba* grow well at 25–30°C in nutrient-rich conditions; however, their growth at higher temperatures is impaired (26), and their survival at lower temperatures (below 15°C) requires rapid lipid synthesis, possibly with extra energy consumption (18). We hypothesized that bacterial endosymbionts help the amoebae overcome the harsh conditions of temperature extremes. We therefore compared the growth speed of aposymbiotic amoebae at three different temperatures (15, 30, and 37°C) (Fig. 2). R18DOX grew in PYG media significantly more slowly than R18WT did at 30°C. In contrast, S13RFP grew in PYG significantly faster than S13WT did at 30°C and 15°C. There was no active growth of S13WT amoebae at 37°C, although a significant increase in growth speed was observed for R18WT. In other words, elimination of endosymbiotic bacteria in two different amoebae adversely influenced their growth speed.

### Decreased motility, but unimpaired pinocytosis and phagocytosis, in aposymbiotic R18 amoebae

**Fig. 3:**
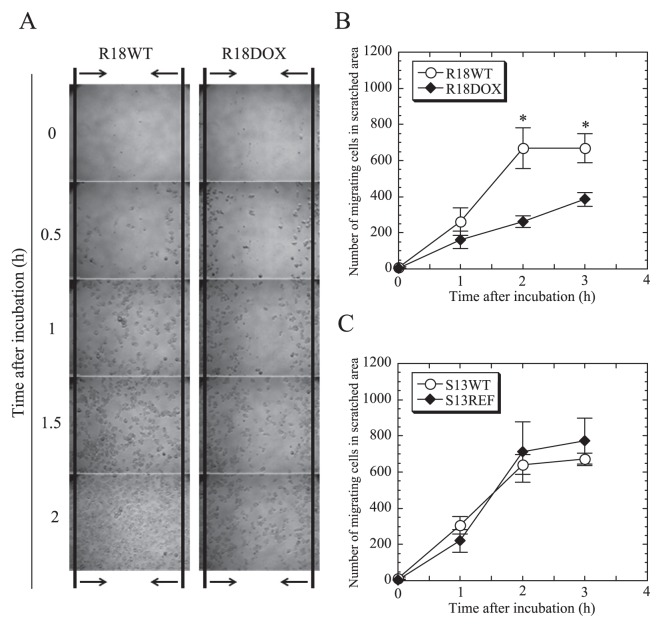
Influence of antibiotic treatment on amoebal motility. (A) Representative motility images were captured for up to 3 h. The space between the arrows indicates the observed area. 100× magnification. (B and C) Comparison of the number of migrating amoebae [R18 amoebae (B), S13 amoebae (C)] in a scratched area. The data show the mean ± SD of at least three independent experiments. *P<0.05 *vs*. cultures with aposymbiotic amoebae (Student’s *t*-test).

It is well known that pathogenic chlamydiae, which are related to environmental chlamydiae, can cause actin remodeling in infected host cells, presumably affecting host cellular motility and division (19). Based on this, we next examined whether the elimination of endosymbionts impaired amoebal motility. As shown in Fig. 3, the motility of R18DOX amoebae was significantly decreased compared with that of R18WT amoebae, although there was no significant change in motility between S13RFP and S13WT amoebae. We also assessed pinocytosis and phagocytosis in amoebae before and after antibiotic treatment. Neither pinocytic nor phagocytic activity was altered in aposymbiotic amoebae compared with in their respective symbiotic amoebae (data not shown). Thus, while eR18 *Protochlamydia* (but not eS13 *Neochlamydia*) altered amoebal motility, the presence of endosymbiotic bacteria in these amoebae did not affect pinocytosis or phagocytosis.

### Comparison of phalloidin staining patterns between aposymbiotic and symbiotic amoebae, and SEM morphological features

**Fig. 4:**
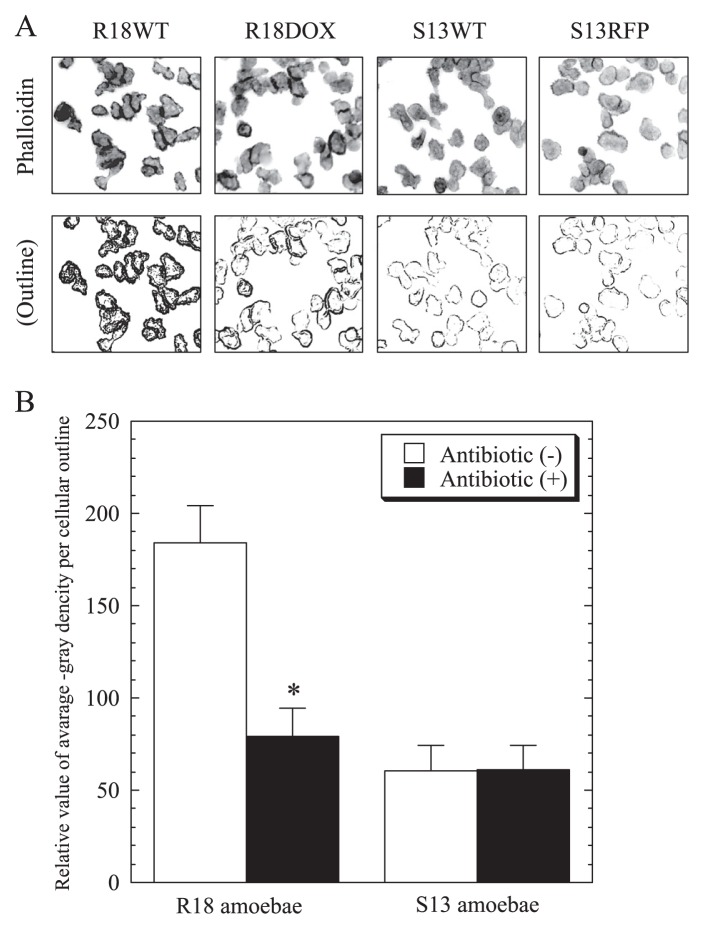
Comparison of phalloidin staining patterns between symbiotic (R18WT and S13WT) and aposymbiotic (R18DOX and S13RFP) amoebae. (A) Representative phalloidin staining images of R18 and S13 amoebae before and after antibiotic treatment (upper panels) and the captured amoebal outline images converted to gray-gradual signals as described in Materials and Methods (lower panels); 200× magnification. (B) The relative value of averaged gray density per amoebal outline was compared between symbiotic and aposymbiotic amoebae. The data show the mean ± SD of at least three independent experiments. *P<0.05 *vs*. the value for the respective aposymbiotic amoebae (Student’s *t*-test).

**Fig. 5:**
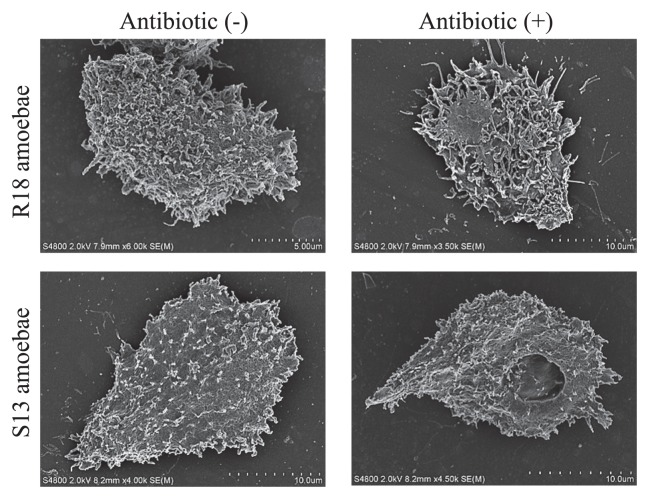
Morphological changes in aposymbiotic amoebae. Representative SEM images of R18 and S13 amoebae before and after antibiotic treatment.

Because a social amoebal (*Dictyostelium*) mutant has been shown to be defective in the Ras pathway related to change of motility through actin remodeling (2, 3), we tested whether the change of motility in R18DOX was associated with alterations in actin localization. We found a significant difference in phalloidin staining patterns between symbiotic and aposymbiotic R18 amoebae, showing a change in actin localization on the amoebal surface (Fig. 4). No such change was present in S13 amoebae, although both S13 symbiotic and aposymbiotic amoebae were more weakly stained than R18 amoebae. Interestingly, we confirmed by SEM that R18 aposymbiotic amoebae irregularly expressed more surface pseudopodia than R18WT symbiotic amoebae (Fig. 5).

### Decreased growth rate, but unimpaired motility, pinocytosis, and phagocytosis in C3 and S13RFP amoebae infected with *Protochlamydia*

**Fig. 6:**
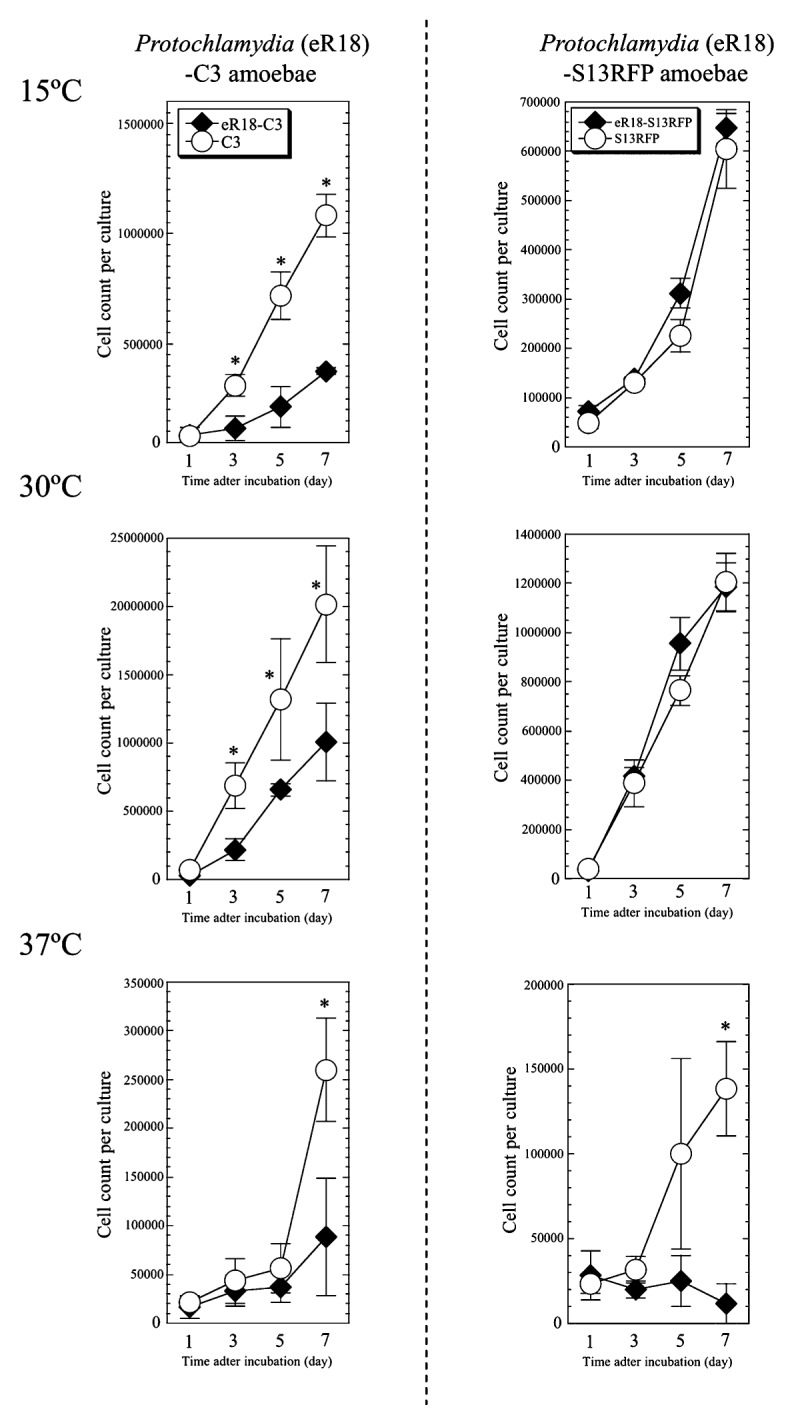
Decreased growth speed in C3 and S13RFP amoebae infected with *Protochlamydia*. The growth speed was assessed as described in Materials and Methods. The data show the mean ± SD of at least three independent experiments. *P<0.05 *vs*. cultures with uninfected C3 amoebae at each time point (Student’s *t*-test).

To test whether these interactions between endosymbiotic bacteria and host amoebae are an inherent phenomenon, we compared the growth speed, motility, pinocytosis, and phagocytosis between C3 and S13RFP amoebae with or without infection with eR18 *Protochlamydia*. Although the motility, pinocytosis, and phagocytosis of the infected C3 and S13RFP amoebae were not altered (data not shown), the growth speed of both infected C3 (Fig. 6 [left]) and S13RFP (Fig. 6 [right]) amoebae was differentially impaired compared with their respective uninfected amoebae. Thus, it is evident that while eR18 *Protochlamydia* can be advantageous to their original host R18 amoebae, these bacteria can harm both C3 and S13 amoebae, neither of which are their original host. *Protochlamydia* infection in C3 and S13RFP amoebae disrupted the host amoebae for up to 2 weeks (data not shown).

### Growth speed and motility of R18DOX was restored by re-infection with eR18 *Protochlamydia*

**Fig. 7:**
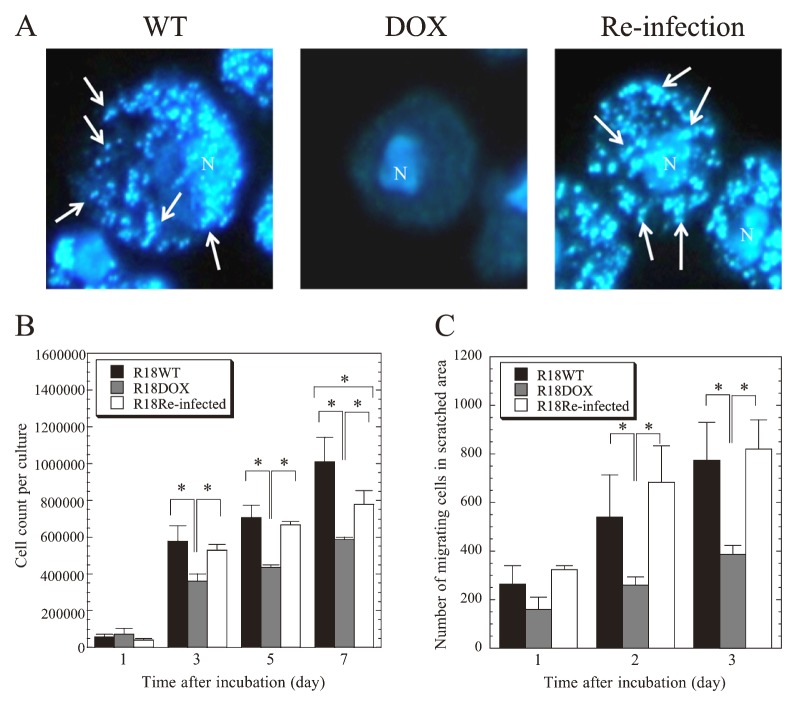
Restored growth speed and motility of R18DOX aposymbiotic amoebae by re-infection with eR18 *Protochlamydia*. (A)Representative DAPI staining images of wild-type R18WT amoebae (WT) R18 aposymbiotic amoebae before (DOX) and after re-infection (Re-infection). Amoebae were observed using a conventional fluorescence microscope at 1,000× magnification. Arrows show representative bacterial clusters. N, amoebal nucleus. (B and C) Effect on growth speed (B) and motility (C) of R18DOX aposymbiotic amoebae of re-infection with *Protochlamydia*. R18DOX aposymbiotic amoebae were re-infected with eR18 *Protochlamydia* at a multiplicity of infection of 10. The data show the mean ± SD of at least three independent experiments. *P<0.05 *vs*. cultures with R18WT amoebae (Student’s *t*-test with Bonferroni correction).

Finally, we analyzed whether re-infection with an endosymbiont could restore the growth speed and motility of aposymbiotic amoebae. While eR18 *Protochlamydia* can reinfect host amoebae, eS13 *Neochlamydia* are not able to do this; therefore, only eR18 endosymbionts and aposymbiotic R18DOX amoebae were used for this experiment. Staining with DAPI confirmed that the prevalence of re-infected amoebae was approximately 100%, similar to that for symbiotic R18WT amoebae, 7 days post-infection. This infection rate was maintained for several months (data not shown). As shown in Fig. 7A, there was no significant difference between the numbers of bacterial clusters observed in R18WT and R18 re-infected amoebae, demonstrating successful re-infection. Furthermore, the growth speed and motility of the re-infected amoebae recovered to preelimination levels (Fig. 7B [growth speed] and C [motility]). The *Protochlamydia* appeared able to rebuild a stable interaction with R18 amoebae.

## Discussion

We used relatively high-concentration antibiotics to establish aposymbiotic amoebae. The concentration was comparable to that used for the elimination of pathogenic chlamydiae, such as *Chlamydia trachomatis* or *C. pneumoniae*, from epithelial cells (6, 12). Nine-day incubation was necessary for complete elimination, permitting stable culture of the aposymbiotic amoebae for at least 5 months. To prevent reconstitution of the bacterial endosymbionts into the amoebae, the amoebae stored at −80°C were freshly cultured and used experimentally within one month. In contrast, it has been reported that the treatment of amoebae with either DOX or RFP, even at a low concentration, was effective at eliminating *Parachlamydia acanthamoeba* (24). This suggests a diversity of antibiotic susceptibilities among environmental chlamydiae.

Why the influence of endosymbionts on the growth speed of amoebae differs between *Protochlamydia* and *Neochlamydia* remains unknown; however, because the difference in growth rate between symbiotic and aposymbiotic amoebae was seen only at 30°C and 37°C, it is possible that bacterial endosymbionts, in particular in the *Protochalmydia*-R18 amoeba interaction, could confer tolerance to heat shock stress on their host amoebae. In fact, it has been reported that the bacterial endonuclear symbiont *Holospora* enhances *hsp70* gene expression in its host ciliate *Paramecium* (13). Thus, the interaction of *Protochlamydia* with R18 amoebae might confer an advantage, allowing the amoebae to survive in warm environments (37°C).

Many studies have indicated that amoebal motility is regulated by actin-mediated events through the activation of Ras small GTPases (2, 3, 33); therefore, the change of motility in R18DOX may be associated with alterations in actin-mediated events. Accordingly, there was a significant difference in actin accumulation between R18 symbiotic and aposymbiotic amoebae. In addition, the presence of many pseudopodia on the surface of aposymbiotic R18 amoebae, possibly associated with fruitless motion such as a loose pulley, supports this. Thus, it is possible that the endosymbiotic bacteria eR18 *Protochlamydia* alter amoebal motility by actin-mediated processes such as actin remodeling. It remains unknown why there was a difference in surface actin accumulation between R18WT and S13WT amoebae. Meanwhile, because another amoeba, *Dictyostelium*, with a disrupted *rasS* gene that regulates actin-mediated events, displays defects in pinocytosis and phagocytosis (2, 3), the lack of either pinocytosis and phagocytosis changes in R18DOX aposymbiotic amoebae was unexpected. The reason for this is unknown; however, it is possible that the morphological alterations occurred in only a limited area, which did not affect the overall phenotype.

As expected, the growth speed and motility of R18DOX aposymbiotic amoebae re-infected with eR18 *Protochlamydia* recovered to the pre-elimination levels. On the other hand, the growth speed of infected C3 and S13RFP amoebae was significantly impaired compared with that of uninfected amoebae, which results in amoebal lysis (data not shown). Thus, while the *Protochlamydia* work together with their native host R18 amoebae, they have a harmful effect on the non-native hosts C3 and S13. This suggests bacterial adaptation to their host amoebae through mutual evolution. We do not yet have a definitive explanation of the benefits to both the bacterial endosymbionts and amoebae that contribute to maintaining stable symbiosis, and they may vary depending on the species involved: *Protochlamydia* may confer an advantage to R18 amoebal predation, and *Neochlamydia* might provide a stable food source or part of an organelle for S13 amoebae.

In conclusion, our data have shed new light on the functional interactions and co-evolutionary relationships among microbes. We have demonstrated that the presence of endosymbiont eR18 *Protochlamydia* confers advantages related to growth speed and motility to R18 amoebae, but that the presence of eS13 *Neochlamydia* does not. The detailed genetic mechanisms remain unknown, and comprehensive genomic studies are therefore needed to understand the complex interactions between environmental chlamydiae and amoebae.
